# Correlation Analysis between Retention of Gd-DTPA in the Cystic Area of Brain Metastasis and MRI Signs

**DOI:** 10.1155/2022/2738892

**Published:** 2022-06-18

**Authors:** Jili Wang, Shanshan Qu, Qinyan Xu, Zhaofeng Jin, Tian Li, Shuxian Zhang, Xihe Sun

**Affiliations:** ^1^Department of Medical Imaging, Weifang Medical University, Weifang 261653, China; ^2^Imaging Department, Shouguang People's Hospital, Shouguang 262700, China; ^3^Ultrasonic Department, Weifang People's Hospital, Weifang 261000, China; ^4^Imaging Department, Affiliated Hospital of Weifang Medical University, Weifang 261653, China; ^5^Department of Clinical Psychology, Weifang Medical University, Weifang 261653, China; ^6^School of Basic Medicine, Fourth Military Medical University, Xi'an 710032, China

## Abstract

**Objective:**

The aim of this study is to investigate gadolinium-diethylenetriaminepentacetate (Gd-DTPA) retention in the cystic area of brain metastasis and its correlation with MRI signs.

**Methods:**

Clinical and MRI data of 76 patients with brain metastasis in the cystic area were collected. The contrast signal intensity (CSI) of the cystic area and edema area in the plain scan, enhanced scan, and plain scan after enhancement within 1 month (hereafter referred to as “enhanced plain scan”) were analyzed to determine whether Gd-DTPA was retained in these areas. The lesions with higher CSI values on the enhanced plain scan were classified as the Gd-DTPA retention group and the remaining lesions as the Gd-DTPA-free group. The two groups were compared to determine significant differences in primary lesion type, tumor size, tumor location, capsule wall thickness and morphology, peritumoral edema, and renal function.

**Results:**

A total of 123 lesions were detected. The CSI of the enhanced plain scan exceeded that of the plain scan and enhanced scan in the cystic area (*P* < 0.05). There were 54 lesions (43.9%) with Gd-DTPA retention in the cystic area and 69 lesions (56.1%) without Gd-DTPA retention. Significant differences were observed in tumor size and cystic wall thickness between the two groups (*P* < 0.05), while no significant differences in primary lesion type, cystic wall shape, peritumoral edema, or function were observed.

**Conclusion:**

The retention of Gd-DTPA was found in the cystic area of some brain metastases, which was correlated with tumor size and cystic wall thickness.

## 1. Introduction 

Brain metastasis (BM) is the major intracranial malignancy in adults, often occurring in patients with lung cancer, breast cancer, and melanoma. Patients with primary lung cancer have the highest incidence of BM [1–3], and it has been reported worldwide that the incidence ranges from 23% to 65% [4]. Enhanced magnetic resonance imaging (MRI) is currently recognized as the most sensitive test to detect BM [5].

The occurrence of necrotic cystic lesions in BM is commonly observed in clinical cases; however, very few studies in China and abroad have reported on the biological characteristics of cystic lesions. To date, only one case has been reported internationally in which the signal intensity was found to increase in the BM cystic area after a delay of 24 h [6]. In China, only one study confirmed that gadolinium-diethylenetriaminepentacetate (Gd-DTPA) was retained in the cystic area of some patients with BM following brain MRI with Gd-DTPA enhancement [7]. However, this previous study had a small sample size and a short observation period; moreover, the study did not investigate whether the type of primary tumors, renal function, or MRI signs of tumors such as size, grade, and capsule wall thickness could affect Gd-DTPA retention in the cystic cavity.

To further confirm the existence of Gd-DTPA in BM cystic lesions, our study was conducted with an increased sample size and extension of the observation time to determine the correlation between this phenomenon and MRI signs of tumors, to preliminarily analyze the mechanism underlying this phenomenon and the metabolic status of BM cystic lesions, and to suggest possible approaches for the clinical treatment of cystic BM.

## 2. Materials and Methods

### 2.1. Clinical Data

Clinical data were collected from patients who underwent craniocerebral MRI examination in the Affiliated Hospital of Weifang Medical University from January 2013 to June 2021 and were retrospectively analyzed. The following inclusion criteria were considered: (1) MRI enhanced scan showed necrotic cystic degeneration and annular enhancement in BM, (2) history of primary tumor and pathological results, and (3) plain scan after enhancement was acquired after cranial MRI plain scan and enhanced scan within 1 month, with no history of undergoing MRI enhanced scan in any other hospital.

The data of 76 patients with BM necrosis (45 males and 31 females), ranging in age from 47 to 83 years (65.6 ± 8.4 years), were collected. The primary tumor types included small cell lung carcinoma (30 cases), nonsmall cell lung carcinoma (37 cases), and nonlung cancer (nine cases; one case each of cervical cancer, prostate cancer, gastric cancer, nasopharyngeal cancer, renal cancer, and esophageal cancer and three cases of breast cancer).

This study was approved by the Ethics Committee of the Affiliated Hospital of Weifang Medical University. The requirement to obtain informed consent from the subjects was exempted (Approval number: wyfy-2021-ky-111).

### 2.2. MRI Examination Method

All MRI data were acquired by a 3.0T MRI scanning system (Signa HDxt, GE Healthcare, Waukesha, WI, USA), which was equipped with an eight-channel phase array head coil. A normal cranial brain scan was acquired first in the supine position with the head placed on the front. Gd-DTPA was then used as a contrast enhancement agent and injected through the anterior elbow vein with a high-pressure syringe at the dose of 0.1 mmol/kg; the injection rate was 3 mL/s. Finally, the elbow vein was rinsed with the same amount of normal saline.

For conventional plain scanning in the axial position, we used the following scanning sequence and parameters: T1WI (TE 24 ms, TR 2250 ms, FOV 240 mm, layer thickness 6 mm, layer interval 1.5 mm, matrix 320 × 192, NSA 1), T2-flair (TE 165 ms, TR 8575 ms, FOV 240 mm, layer thickness 6 mm, layer interval 1.5 mm, matrix 256 × 160, NSA 1), and susceptibility-weighted imaging (SWI; TE 35 ms, TR Minimum, FOV 240 mm, matrix 512 × 192). For enhanced scanning in the axial position, we used the following scanning sequence and parameters: T1WI (TE 24 ms, TR 1350 ms, FOV 240 mm, layer thickness 6 mm, layer interval 1.5 mm, matrix 320 × 160, NSA 1).

### 2.3. Image Analysis

GE AW4.5 workstation software was used to select the largest scan area and a uniform signal layer of each lesion in combination with the performance of each lesion in three scans; the region of interest with a size of 5−10 mm^2^ was delineated in each scan. The signal intensity of each BM cystic area, edema area, and contralateral normal white matter area was measured three times. The average value was recorded as the signal intensity (SI) of the region, and the contrast signal intensity (CSI) of the cystic lesion area and the surrounding edema area of the BM was calculated according to the following formula: CSI = SIL/SIN, where SIL represents the signal intensity of the lesion and SIN represents the signal intensity of the contralateral normal white matter area.

In the picture archiving and communication system, two MRI clinicians with over 5 years of experience in scanning and interpreting MR images of the central nervous system reviewed the images in a double-blind manner. The number of lesions, tumor size, location of tumor occurrence, thickness and shape of the cyst wall, degree of peritumoral edema, enhancement method, and signal characteristics of each case were observed, and the conclusions were drawn accordingly. In the case of any disagreement, the opinion of a deputy chief physician was sought to reach a consensus.

### 2.4. Observation Index

1. Tumor size: from the T1WI enhanced scan image, the diameter of the maximum cross section of the tumors was measured on the axial image, and the tumors were classified into three levels according to the size: grade I: <1.0 cm, grade II: between 1.0 and 3.0 cm, and grade III: >3.0 cm.
2. Location: BM can be classified according to the location of occurrence: frontal lobe, parietal lobe, temporal lobe, occipital lobe, cerebellum, etc.; it can also be classified into supratentorial and infratentorial. In the present study, we classified BM according to the blood supply areas: the internal carotid artery and the vertebrobasilar artery. As the lesion grew across the lobes, the location of the lesion with the largest proportion of volume was recorded.
3. Cystic wall thickness [8, 9]: the thickest layer of the cystic wall was selected from the T1WI enhanced scan image, and a straight line was drawn from the outer edge of one lesion to the other side. The length of the straight line between the outer edge of the lesion and the inner wall was measured, and the thickness of the cystic wall was equal to the difference between the outer edge and the inner wall length. The lesions were divided into thin (≤5 mm) and thick (>5 mm) according to the measured thickness of the cystic wall.
4. Cystic wall morphology [10]: from T1WI enhanced images, BM was divided into three types based on the morphology of the cystic wall: smooth, nonsmooth, and nodular. Smooth: the cystic wall showed uniform thickness, regularity, smooth inner wall, clear boundary, and uniform ring enhancement on enhanced scan. Nonsmooth: the cystic wall was characterized by uneven thickness and roughness, and the inner wall was incomplete. The enhanced scan showed circular uneven enhancement. Nodular: the cystic wall was partially incomplete, and visible wall nodules could be observed in the cystic wall. The cystic wall and wall nodules were significantly enhanced in the enhanced scan.
5. Degree of peritumoral edema [11]: combined with T2WI-FLAIR and T1WI enhanced scanning images, the layers with the largest area of the tumor and peritumoral edema were selected, and the longest diameter of the tumor and degree of peritumoral edema were measured. The degree of peritumoral edema was categorized into four grades: no edema: no obvious edema zone around the tumor, mild edema: the maximum diameter of peritumoral edema was half of the maximum diameter of the tumor, moderate edema: the maximum diameter of peritumoral edema was between half of the maximum diameter of the tumor and the maximum diameter of the tumor, and severe edema: the maximum diameter of peritumoral edema exceeded the maximum diameter of the tumor.
6. Renal function grading: renal function was classified into the following categories according to endogenous creatinine clearance rate (CCr): normal: CCr > 90 mg/dL, mild injury: 60 mg/dL < CCr ≤90 mg/dL, moderate injury: 30 mg/dL < CCr ≤60 mg/dL, and severe injury: 15 mg/dL < CCr ≤30 mg/dL.

### 2.5. Statistical Analysis

All data were analyzed by SPSS 23.0 statistical software, and the measurement data were tested for normal distribution and subjected to Kolmogorov–Smirnov test. Data with *P* > 0.05 were considered to follow a normal distribution. Normally distributed data were expressed as mean ± standard deviation and compared by analysis of variance (ANOVA). Nonnormally distributed data were expressed as median and interquartile ranges and compared by the Friedman rank-sum test of relevant multiplicity. For statistical significance, the Friedman M test was used for pairwise comparison [12], and the chi-square test or Fisher's exact test was used to analyze count data. Differences with *P* < 0.05 were considered to be statistically significant.

## 3. Results

### 3.1. Comparison of Three CSI Scans in the Tumor Cystic Area and Edema Area

The MRI data of 76 patients with cystic lesions of BM were collected, which included 123 lesions. The data measured in the plain scan of the cystic lesion area and the enhanced scan of the edema area followed normal distribution (*P* > 0.05), while the remaining data showed nonnormal distribution (Table 1). Therefore, the difference among three scanning times of CSI was compared using the relevant multiple Friedman rank-sum test. The CSI in the cystic lesion area showed a significant difference in the three scanning times (*P* < 0.05). No significant difference was observed (*P* > 0.05) in the Friedman M test for pairwise comparison between the plain scan and enhanced scan, and no significant difference was observed (*P* > 0.05), indicating that there was no significant change in the necrotic area signal after enhanced scan. A significant difference (*P* < 0.05) was observed in the comparison between the plain scan after enhancement within 1 month (hereafter referred to as “enhanced plain scan”) and the plain scan alone, indicating that the signal of cystic lesions increased in the enhanced plain scan as compared with that in the plain scan. Moreover, a significant difference (*P* < 0.05) was observed in the comparison between the enhanced plain scan and enhanced scan, thus indicating that the signal of the necrotic area increased in enhanced plain scan as compared with that in the enhanced scan.

**Figure 1:**
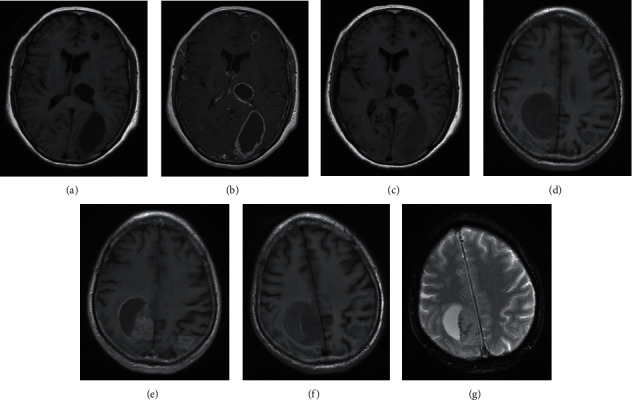
Contrast signal intensity (CSI) differences in the three scanning times. A–C show plain scan T1WI images of the craniocerebral axial position, enhanced scan T1WI, and enhanced plain scan, respectively. The BM cystic area in the left parietal-occipital lobe showed a low signal during the plain scan and enhanced scan, and the signal intensity increased significantly in the enhanced plain scan. D–G show plain scan T1WI images of the craniocerebral axial position, enhanced scan T1WI, enhanced plain scan, and SWI, respectively. The BM cystic area in the right frontal-parietal lobe showed a low signal during the plain scan and enhanced scan, and the signal intensity increased in the enhanced plain scan. Magnetic-sensitive low signals were not observed in the SWI scans to exclude bleeding.

A comparison of the three scanning times in the edema area showed a significant difference (*P* < 0.05). The Friedman M test was performed for pairwise comparison between the plain scan and enhanced scan, and no significant difference was observed (*P* > 0.05), indicating no significant change in the signal of the edema area during the two scans. A comparison between the plain scan and enhanced plain scan showed a significant difference (*P* < 0.05), indicating that the signal of the edema area increased in the enhanced plain scan as compared with that in the plain scan. A significant difference was noted in the comparison between enhanced plain scan and enhanced scan (*P* < 0.05), indicating that the signal of the edema area increased in the enhanced plain scan as compared with that in the enhanced scan (Table 2 and Figure 1).

An analysis of CSI in the three scans of the BM cystic area showed that the signal of the cystic area increased in the enhanced plain scan, whereas no low signal was found in the cystic area in the SWI scan. Excluding the high signal caused by hemorrhage, the infiltration of Gd-DTPA could be considered as the reason for the high signal in the plain scan T1WI after enhancement within 1 month. This implied the presence of Gd-DTPA in the BM cystic area.

### 3.2. Comparison of Taxonomic Variables in the BM Cystic Areas

The CSI of the enhanced plain scan was compared with that of the plain scan and enhanced scan in cystic lesions. The lesions showing higher CSI values than those for the plain scan and enhanced scan were included in the Gd-DTPA retention group (experimental group). This group included 54 lesions, accounting for approximately 43.9% of the total lesions. The remaining lesions were included in the Gd-DTPA-free group (control group); this group included 69 lesions, accounting for 56.1% of the total lesions. Regarding the classification variables, significant differences were observed in tumor size and cystic wall thickness between the two groups (*P* < 0.05). A comparison of tumor size in two groups revealed that the number of grade I tumors in the experimental group was significantly smaller than that in the control group (3.7% vs. 31.9%), and a significant difference was observed (*P* < 0.05). No significant difference was observed in the number of grade II and III tumors between the two groups (*P* > 0.05), but the number of grade II and III tumors in the experimental group was higher than that in the control group (63.0% vs. 47.8% and 33.3% vs. 20.3%, respectively). Moreover, no significant differences were observed in primary tumor type, position, cystic wall morphology, degree of peritumoral edema, and renal function grade (*P* > 0.05) (Table 3).

## 4. Discussion

### 4.1. Interpretation

As a malignant tumor of the CNS with the highest incidence [13], BM occurs in approximately 30%–40% of patients with cancer [14] and is the main cause of morbidity and death in about 20% of these patients [15, 16]. To the best of our knowledge, the present study is the first large-sample study of the correlation between Gd-DTPA retention and MRI signs in the cystic area in China and abroad. A correct understanding of this phenomenon could clarify the confusion of clinicians, and the analysis of the potential mechanism can also enable to better understand the possible metabolism of substances in the BM cystic area, thus providing some insights for treating BM.

### 4.2. Mechanism of Gd-DTPA Retention

MRI enhanced scanning is the best and most commonly used examination method for BM, and gadolinium agent is the most commonly used contrast agent for MRI enhanced examination currently [17]. Gd-DTPA is widely used in clinical practice because of its characteristics of low molecular weight, good hydrophilicity, and short T1 relaxation time in human tissues [18]. There are two main mechanisms of necrotic cystic degeneration in BM. First, because of the rapid occurrence of BM and poor vascular development of the tumor, blood and oxygen cannot sufficiently reach the tumor cells; this affects their proliferation and delays metastasis. Moreover, the central part of the tumor is prone to hypoxia and liquefaction necrosis because of poor blood supply [19]. In severe cases, only one layer of parenchyma is left, which is known as cystic BM [20]. Second, the absence of the blood-brain barrier, vasogenic edema, intertissue fluid accumulation, and other factors in BM lead to necrotic cystic changes in the tumor, and the lack of drainage lymphatic vessels leads to increased colloid osmotic pressure in the cystic cavity, resulting in the formation of cystic fluid [21, 22]. Histologically, brain metastatic lesions are similar to the primary lesion without blood-brain barrier but with extremely high capillary permeability [23]. The high expression of vascular endothelial growth factor (VEGF) in BM can also lead to the formation of immature angiogenesis and further increase vascular permeability [24, 25]. Therefore, Gd-DTPA with a low molecular weight can easily pass through the vascular wall and enter the cystic necrosis area with a high colloid osmotic pressure, resulting in an increased signal in the cystic necrosis area after an enhanced scan. In the present study, 54 lesions in the cystic area showed an increased signal in the enhanced plain scan, that is, retention of Gd-DTPA in the BM cystic area. The peritumoral edema of BM is vasogenic edema, which is caused by cancer plug blockage, vascular reflux compression, and secretion of tumor active factors by tumor cells, and cases of moderate and severe peritumoral edema are commonly observed among these patients [26, 27]. Previous studies [28] have shown that the generation of peritumoral edema is associated with the expression of aquaporin (AQP) and interleukin-6 (IL-6), especially the high expression of AQP-4 in brain tissues around the BM [29]. In the present study, some cases showed an increased signal in the plain scan after enhancement in the area of the peritumoral edema, which was speculated to be caused by the absence of the blood-brain barrier in the BM, abnormal AQP expression, high capillary permeability, and other factors that affected the metabolism in the edema area.

### 4.3. Differences in Classification Variables between the Two Groups

Significant differences were observed in tumor size and cyst wall thickness between the two groups in the classification variables of the present study. Our results also indicate that Gd-DTPA is more likely to be retained in the BM cystic area with a large tumor size. A Chinese study [30] showed the size of the BM was closely related with the number of blood vessels around the lesions. The number of peritumoral blood vessels is low when the tumor is small, and blood vessels remain adjacent to the tumor but do not penetrate it. Small peritumoral vessels are formed gradually close to the tumor and become tumor blood vessels with the growth of the tumor. The larger is the tumor size, the denser the peritumoral blood vessels. The peritumoral blood vessels that penetrate into the tumors are the main blood vessels, which finally grow into a dense vascular network of trophozoites [31, 32]. Moreover, the larger is the volume of metastases, the more likely the peritumoral vessels to be damaged [33]. In such cases, gadolinium ions enter the necrotic area of the tumor through the damaged peritumoral blood vessels. Therefore, the retention of Gd-DTPA in the BM cystic area is correlated with tumor size.

Among the 18 grade III tumors in the experimental group, 14 tumors (77.8%) had a thick wall. In the control group, there were only seven tumors (50%) with a thick wall, which was significantly lower than that in the experimental group. BM mainly comprises cystic fluid and cystic wall, in which the cystic fluid is mainly derived from the dissolution, destruction, and active secretion of tumor cells and the filtration products of plasma after the destruction of the blood-brain barrier, whereas the cystic wall is mainly composed of a large number of blood vessels, tumor cells, connective tissue, and reactive glial tissue [34]. The diameter of the vascular lumen increases, which further increases the damage of blood vessels because of thickening of the capsule wall and higher vascular density. Consequently, more gadolinium contrast agent enters the internal BM cystic necrosis zone through damaged blood vessels and increases the signal. Based on these findings, it can be concluded that the thicker the capsule wall of BM, the more likely the retention of Gd-DTPA in the cystic area.

### 4.4. Limitations

The present study had the following limitations: (1) the number of included cases with primary foci of nonlung cancer was small, and the discussion of BM cystic lesions from different tissue sources was inadequate, (2) few cases of renal function injury were included, and (3) in-depth correlation analysis between renal injury and the administration of the gadolinium contrast agent was not performed.

In conclusion, Gd-DTPA was found in some BM cystic lesions in plain scans after MRI enhanced scans, which was correlated with tumor size and cystic wall thickness. It is expected that by investigating the time period of discharge of the gadolinium contrast agent in cystic lesions, more effective therapeutic approaches for treating BM can be developed. In the future, we may develop drugs with molecular properties similar to those of gadolinium ions or drugs with gadolinium ions as carriers to better achieve the treatment of BM by studying the metabolic clearance of gadolinium agents in the cystic area and the duration of their metabolism.

## Figures and Tables

**Table 1:** Normal and nonnormal distribution of the MRI data of the cystic area and edema area in patients with BM.

	Skewness	Kurtosis	*P*
Cystic area			
Plain scan	1.387	5.723	0.200
Enhanced scan	0.480	−0.928	<0.001
Enhanced plain scan	0.393	1.601	0.024

Edema area			
Plain scan	1.429	5.844	0.032
Enhanced scan	−0.657	1.339	0.200
Enhanced plain scan	−0.556	0.589	0.001

**Table 2:** Comparison of CSI values in the cystic area and edema area in patients with BM.

	Median	Interquartile range	*χ* ^2^	*P*
Cystic area			9.577	0.008
Plain scan	0.477	0.357		
Enhanced scan	0.349	0.535		
Enhanced plain scan	0.664	0.228		

Edema area			28.309	<0.001
Plain scan	0.809	0.250		
Enhanced scan	0.857	0.231		
Enhanced plain scan	0.899	0.209		

*Note.* The multiple Friedman M test showed differences in the CSI values of cystic lesions and edema areas among the three scans. Pairwise comparison of Friedman M tests was performed: (1) cystic lesions: no significant difference was observed between the plain scan and enhanced scan (*P*=1.000), a significant difference was noted between the plain scan and enhanced plain scan (*P*=0.018), and a significant difference was observed between the enhanced scan and enhanced plain scan (*P*=0.027); (2) edema area: no significant difference was observed between the plain scan and enhanced scan (*P*=0.091), a significant difference was noted between the plain scan and enhanced plain scan (*P* < 0.001), and a significant difference was observed between the enhanced scan and enhanced plain scan (*P*=0.005).

**Table 3:** Comparison of classification variables for the BM cystic area between the two groups (*n* (%)).

Relevant factors	Experimental group (*n* = 54)	Control group (*n* = 69)	*χ* ^2^	*P*
Primary lesion type			3.832	0.147
Small cell lung cancer	19 (35.2)	31 (44.9)		
Nonsmall cell lung cancer	31 (57.4)	28 (40.6)		
Nonlung cancer	4 (7.4)	10 (14.5)		

Tumor size			15.584	<0.001
Grade I	2 (3.7)^a^	22 (31.9)		
Grade II	34 (63.0)^b^	33 (47.8)		
Grade III	18 (33.3)^b^	14 (20.3)		

Position			0.067	0.796
Internal carotid artery blood supply area	31 (57.4)	38 (55.1)		
Vertebrobasilar artery blood supply area	23 (42.6)	31 (44.9)		

Capsule wall thicknesses			6.071	0.014
Thin	20 (37.0)	41 (59.4)		
Thick	34 (63.0)	28 (40.6)		

Cystic wall morphology			0.238	0.888
Smooth type	16 (29.6)	23 (33.3)		
Nonsmooth type	14 (25.9)	18 (26.1)		
Nodular type	24 (44.5)	28 (40.6)		

Degree of peritumoral edema			3.922	0.252 ^*∗*^
No edema	2 (3.7)	0 (0)		
Mild	16 (29.6)	23 (33.3)		
Moderate	20 (37.1)	19 (27.6)		
Severe	16 (29.6)	27 (39.1)		

Renal function			2.510	0.113
Normal	41 (75.9)	60 (86.9)		
Mild impairment	13 (24.1)	9 (13.1)		

*Note.* Compared to the control group, ^a^*P* < 0.05, ^b^*P* > 0.05; ^*∗*^Fisher's exact probability method.

## Data Availability

Data are available from the last author upon reasonable request.
